# Heating degree day spatial datasets for Canada

**DOI:** 10.1016/j.dib.2023.109450

**Published:** 2023-07-26

**Authors:** Heather MacDonald, John Pedlar, Daniel W. McKenney, Kevin Lawrence, Kaitlin de Boer, Michael F. Hutchinson

**Affiliations:** a Great Lakes Forestry Centre, Canadian Forest Service, Natural Resources Canada, P6A 2E5 1219 Queen Street East, Sault Ste. Marie, Ontario, Canada; b Fenner School of Environment and Society, Australian National University, Australia

**Keywords:** Grids, Raster, Temperature, Spatial datasets, Heating degree days, HDD, Historical, Thin plate spline, Climate, ANUSPLIN, Canada

## Abstract

Heating degree days (HDD) represent a concise measure of heating energy requirements used to inform decision making about the impact of climate change on heating energy demand. This data paper presents spatial datasets of heating degree days (HDD) for Canada for two thirty-year periods, 1951–1980 and 1981–2010, using daily temperature gauge observations over these time periods. Stations with fewer than nine missing days in a year and greater than nine years of data over each thirty-year period were included, resulting in 1339 and 1679 stations for the 1951–1980 and 1981–2010 periods respectively. Mean absolute error (MAE) of the spatial models ranged from 124.2 Celsius degree days (C-days) for the 1951–1980 model (2.4% of the surface mean) to 137.6 C-days for the 1981–2010 model (2.7%). This note presents maps illustrating cross validation errors at a set of representative stations. The grids are available at ∼2 km resolutions.

**Specifications Table**

**Table:** 

Subject	Earth and Planetary Sciences
Specific subject area	Thin plate spline datasets for heating degree days, Canada, spatial dataset, 1981–2010 & 1951–1980
Type of data	Geospatial grids
How data were acquired	Environment and Climate Change Canada (ECCC) provided daily minimum and maximum temperature values at meteorological stations across Canada (1950–2010).
Data format	Raw – delimited text/ascii Analysed – delimited text/ascii Final - geotiff
Description of data collection	Climate data in Canada are collected through a system of weather stations distributed unevenly across the country. Daily minimum and maximum temperature values from 1339 (1951–1980) and 1679 (1981–2010) weather stations were used to calculate HDD values for 1951–1980 and 1981–2010, which were then interpolated and mapped using ANUSPLIN via tri-variate thin-plate splines.
Data source location	Canada
Data accessibility	https://osf.io/xkpc7/

## Value of the Data

• These datasets were developed in part to support updates to tax credits for northern and isolated areas in Canada for the Canadian Finance Department [1].
• Energy analyses rely on HDD to track changes in natural gas and other energy usage. Historical change in HDD is an important factor in energy consumption planning, particularly in northern areas.
• Users can use the dataset to obtain information about heating requirements for any location in Canada for two long-term periods, 1951–1980 and 1981–2010.
• This data description also provides a case study using published output from ANUSPLIN thin-plate spline program [2].

## 1 Objective

The ‘degree day’ method is used to calculate the difference between mean daily temperature and any given threshold – typically these differences are summed over a period of interest to provide a measure of heat or cold accumulation through time [3]. Heating degree days (HDD) sum the degree to which average daily temperatures are below the temperature of human comfort, defined as 65°F [4], or in Canada as 18 °C [3], [4], [5], [6]. HDD have been analysed to estimate changes in energy usage [7], impacts of climate change [8], [9], and historical trends [9] with respect to how often and how hard a furnace must work to keep a house warm.

The purpose of this brief report is to introduce HDD datasets for Canada for the 1951–1980 and 1981–2010 periods. These datasets were developed in part to support updates to tax benefits for northern and isolated areas in Canada which experience higher than average heating costs in Canada [1]. We describe these datasets and report on the quality and accuracy of the spatial datasets.

## 2 Data Description

### 2.1 Heating degree day (HDD) datasets

**Fig. 1:**
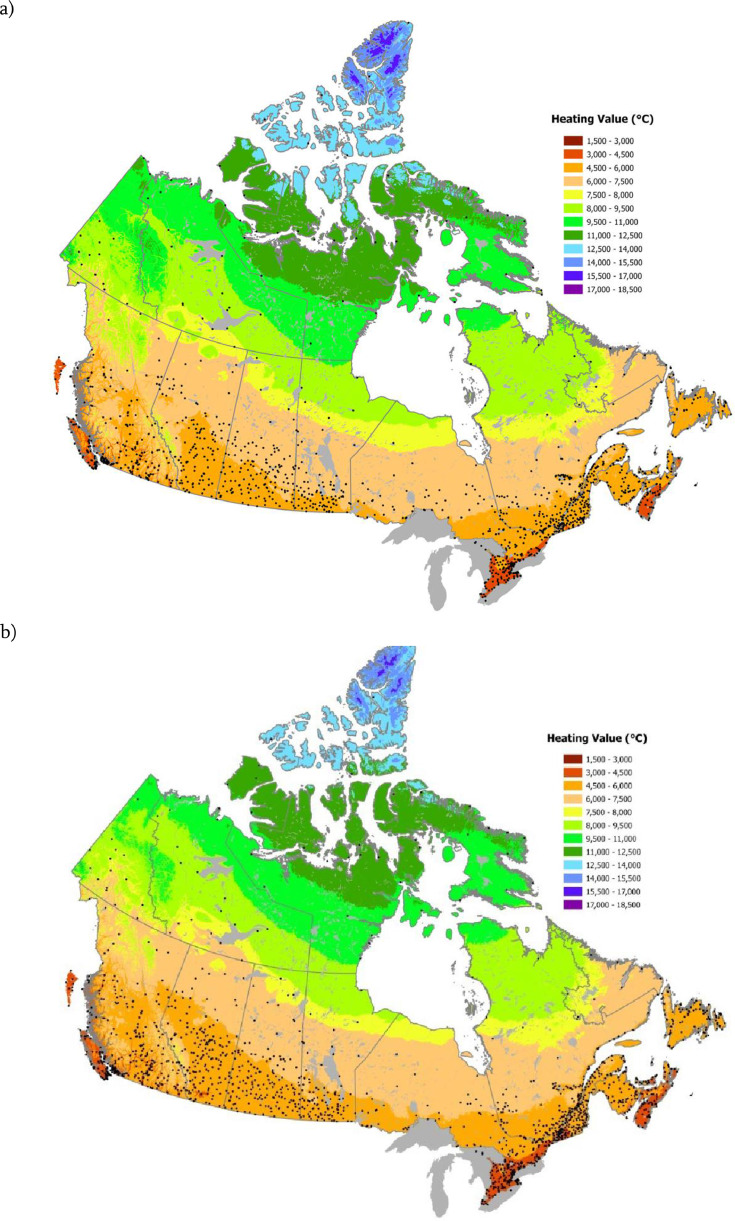
Heating Degree Days for the 1951–1980 (a) and 1981–2010 (b) periods. Black dots show locations of meteorological stations used to develop the thin plate splines.

Canada-wide Heating Degree Day (HDD) gridded datasets were generated for two thirty-year periods, 1951–1980 and 1981–2020 (Fig. 1), using tri-variate thin-plate splines in ANUSPLIN [2] version 4.5 employing a 60′ sec (approximately 2 km) Digital Elevation Model [10].

The datasets documented include:

1. Heating Degree Day Data Files containing Heating Degree Day values calculated for in situ temperature monitoring stations (see [11] detailing a rationale for a similar methodology). HDD, defined as the annual sum of the positive differences between the base temperature of 18 °C and daily temperature, was calculated using the average of maximum and minimum daily temperature according to the following formula:
  (1) $$\text{HDD}=\sum_{i}\left(\left(\theta_{b}-\left(\left(\theta_{\text{MAX}}+\theta_{\text{MIN}}\right)/2\right)\right)\right)$$
  where *i* is the day of the year, *θ_MAX_* is the daily maximum temperature, *θ_MIN_* is the daily minimum temperature, *θ_b_* is the base temperature (18 °C), and *θ_b_ > (θ_MAX_ + θ_MIN_)/2*.
  Raw data file containing minimum and maximum temperatures by station:
  https://osf.io/x397p
  Average HDD calculated for the following 30-year periods:
  1951–1980 (1339 stations): https://osf.io/he3w8
  1981–2010 (1679 stations): https://osf.io/x62vu
  The format used to read in these .dat files is provided at:
  https://osf.io/5m7d8
  COMBINED 1951–1980 and 1981–2010 Heating Degree Day Values (.xlsx format):
  https://osf.io/7u8wz
  1951–1980 and 1981–2010 HDD average values for stations with greater than 10 years of data and the count of number of years of observation data for 1951–1980 and 1981–2010.

2. Output from ANUSPLIN (Lis Files) – 1951–1980 and 1981–2010 “Lis” files contain Station coordinates (latitude, transformed longitude and transformed elevation), HDD value for the station, the fitted value (“Fitted_estimate”), and the individual cross validated values (“CV_estimate”) see [2] for a description of ANUSPLIN output).
  Lis files:
  1951–1980: https://osf.io/29vk4
  1981–2010: https://osf.io/5wyj6
  A genericized script to read in the “Lis files” is provided at:
  https://osf.io/5m7d8
3. Geotiff files – Canada-wide HDD surfaces
  1951–1980: https://osf.io/2zu5p
  1981–2010: https://osf.io/sb5p3

### 2.2 Predictive error of ANUSPLIN datasets

ANUSPLIN produces individual station cross-validation (CV) estimates (“CV_Estimate”), which were compared to HDD calculated from station observations. The CV estimates are individually cross-validated values [2]. Mean error (ME) was calculated using the CV estimate minus calculated HDD. ME and Mean Absolute Error (MAE) are presented in C-days as well as a percentage of the surface mean.

ANUSPLIN CV estimates were biased on average by less than 1C-days for both periods (Table 1). Mean absolute error (MAE) of the ANUSPLIN models ranged from 124.2C-days for the 1951–1980 model to 135.3C-days for the 1981–2010 model. The average MAE for the 1981–2010 period represented 2.7% of the surface mean compared to 2.4% for the 1951–1980 period.

**Table 1:** HDD ME and MAE for 1951–1980 and 1981–2010 30-year periods in C-days and as a % of the surface mean.

Time Period	N	ME in C-days (% of Surface Mean)	MAE in C-days (% of Surface Mean)
1951–1980	1339	0.00 (0.0%)	124.2 (2.4%)
1981–2010	1679	−0.47 (0.0%)	137.6 (2.7%)

**Fig. 2:**
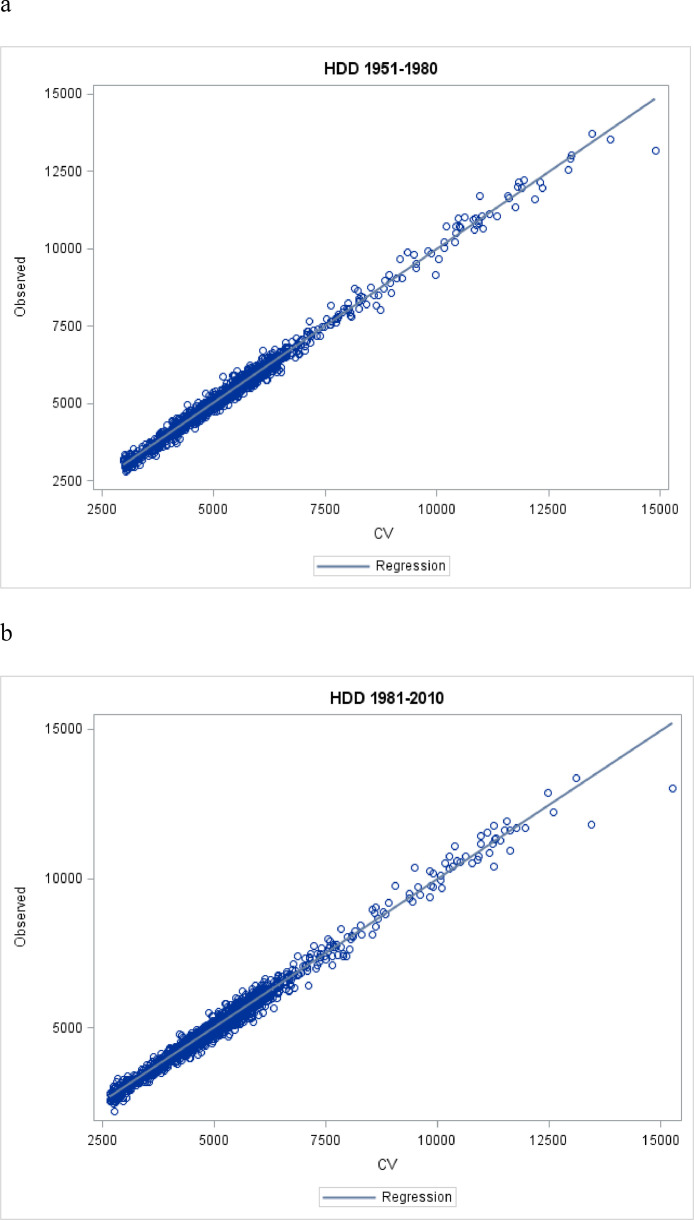
Observed versus CV Estimates for the 1951–80 (a) and 1981–2010 (b) periods.

Plots of observed versus predicted values exhibited strong linear relationships with few outliers for both time periods (Fig. 2).

**Fig. 3:**
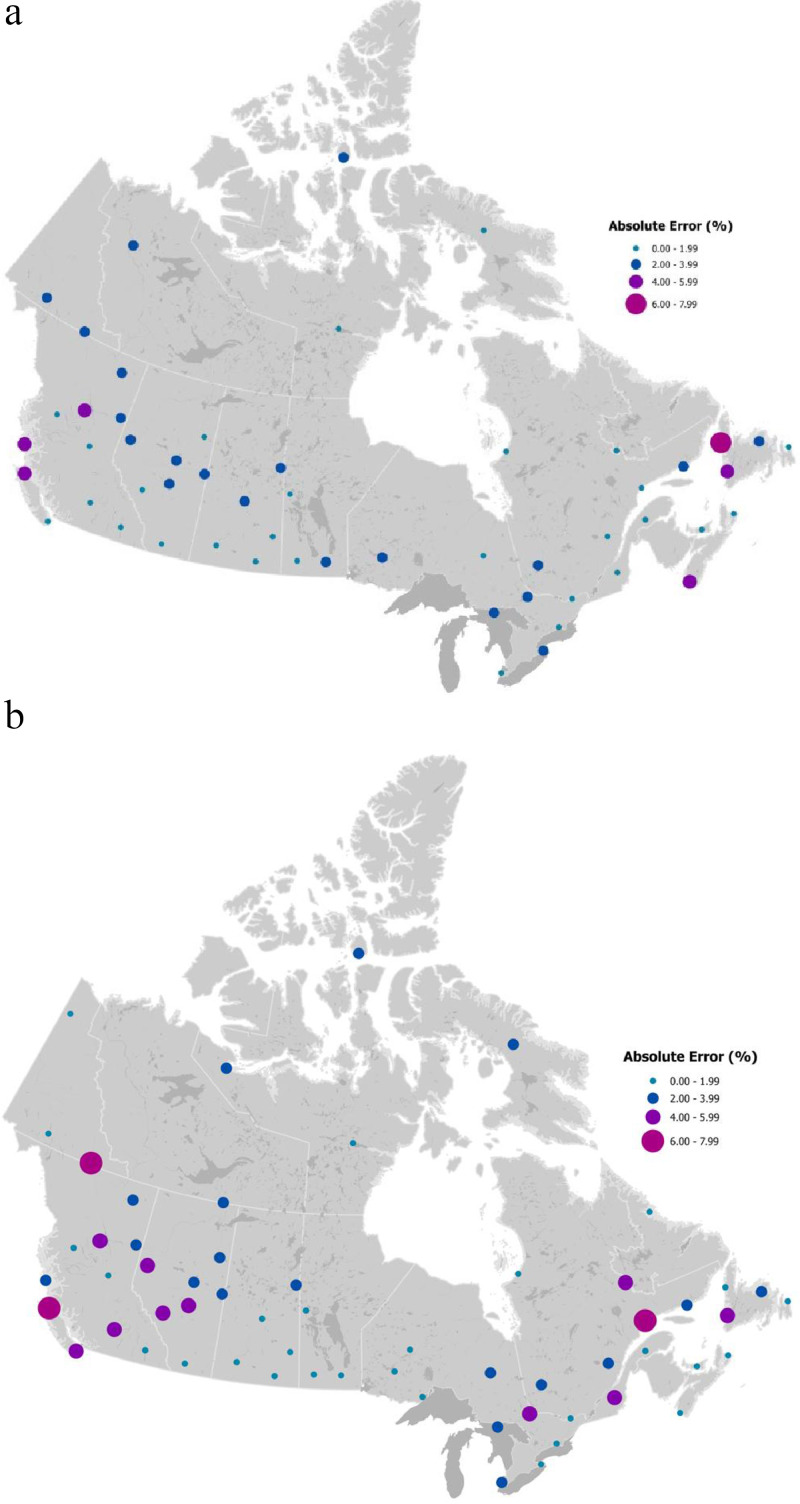
Absolute prediction errors for 60 selected stations for 1951–1980 (a) and 1981–2010 (b) periods.

Predictive errors were plotted for 60 stations selected in previous Canadian studies to better reflect the range in latitude, longitude, and elevation across the country [12,14] as compared with the full set of stations, which are concentrated in southern Canada. Of these 60 stations, 56 stations met the criterion for inclusion in this analysis. Predictive errors at 56 selected stations (Fig. 3) were generally highest in mountainous and coastal regions. Higher errors in areas of complex terrain and coastal areas reflects known challenges with generating spatial models in these highly variable environments for sparse in-situ networks [12], [13], [14]. As a percentage, errors were greater in the 1981–2010 period compared to the 1951–80 period.

## 3 Experimental Design, Materials and Methods

### 3.1 Data acquisition

Environment and Climate Change Canada (ECCC) provided daily minimum and maximum temperature values at meteorological stations across Canada from 1950 to 2010 [15].

### 3.2 Data pre-processing

**Fig. 4:**
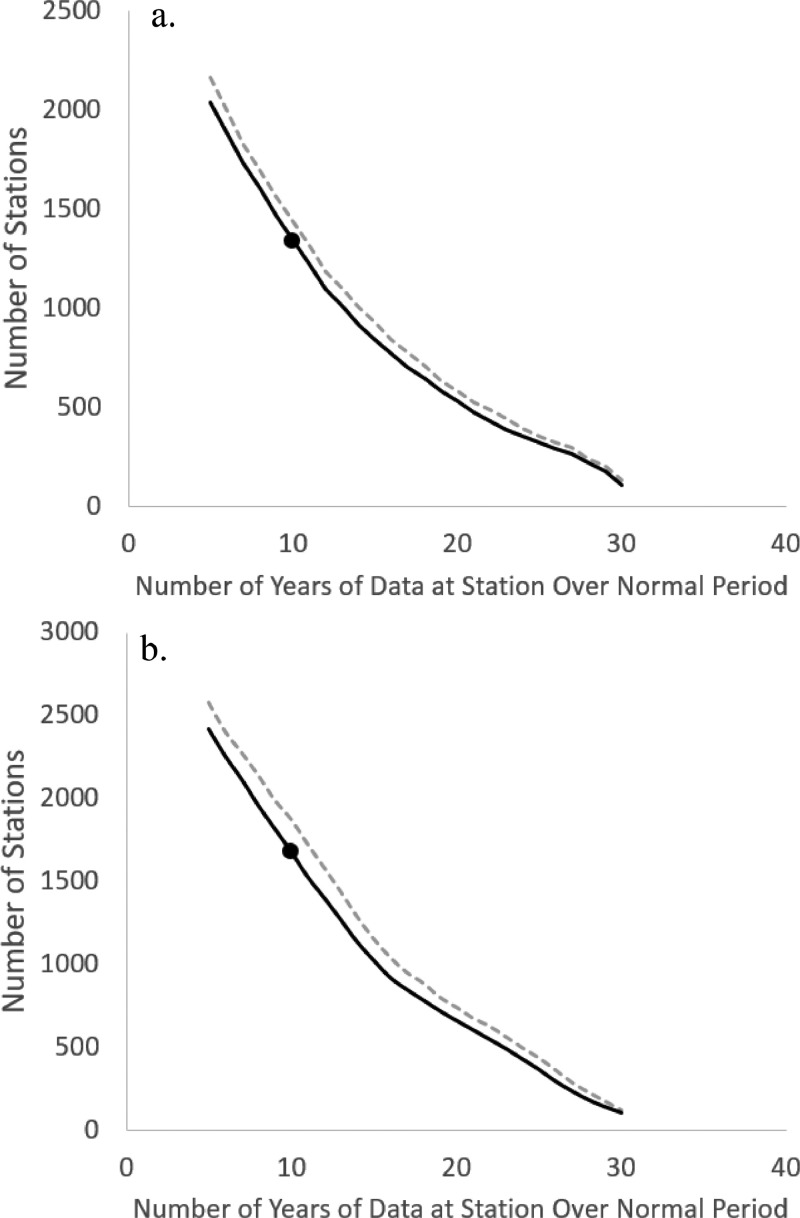
Relationship between the number of years of station data and the number of stations included in the analysis for the a) 1951–80 and b) 1981–2010 normal periods. The solid and dashed lines were generated using only station-years with number of missing days ≤ 10 and ≤ 20 respectively. Cut-offs of 10 missing days per year and 10 years per normal period were used to define the station network employed in the current study (final station numbers shown by black dots).

Plots were generated to examine the number of stations available for analysis based on cut-offs associated with the number of missing days in a year and the number of missing years in a normal period (Fig. 4). We selected stations with ≤ 10 missing days in a year and ≥ 10 years in a normal period for the spatial modelling. With these cut-offs, 1339 and 1679 stations were available for analysis in 1951–1980 and 1981–2010 respectively.

### 3.3 Specifics of implementation

Spatial models were developed in ANUSPLIN [2] and resolved into map form using a 60′ sec (approximately 2 km) DEM [10]. The ANUSPLIN grid was created using latitude, longitude (multiplied by 0.64279), and elevation (multiplied by 1000) as predictors. ANUSPLIN fits partial thin plate smoothing splines constructed from a set of “knots” to noisy multivariate data. A portion of the available observations (in this case, 40%) are selected to limit the complexity of the fitted surface; however, all data points are used to calculate the fitted surface [2].

### 3.4 Experimental results

In addition to predictive error, the quality of the spatial datasets was evaluated using two diagnostic statistics output by ANUSPLIN:

(a) The ratio of the “signal” (S), which ranges between zero and the number of stations (or ‘knots’) selected by ANUSPLIN (nKTS), to the number of knots (S:nKTS). Ratios between 0.2 and 0.8 are considered acceptable [2,12]. HDD dataset ratios of 0.48 and 0.57 (Table 2) were non-problematic.

**Table 2:** HDD Signal to number of knots (S:nKTS) ratio and root GCV for 1951–80 and 1981–2010 30-Year periods.

Time Period	Surface Mean (in C-days)	S:nKTS	RtGCV C-days (% of surface mean)
1951–1980	5400	0.57 (382:669)	147 (2.7%)
1981–2010	5115	0.48 (406:839)	167 (3.3%)
(b) Root GCV (RtGCV). The GCV (Generalized Cross Validation) is calculated by removing each data point and summing the square of the difference of each omitted data point from a surface fitted to all remaining data points [16]. RtGCV, the square root of the GCV, essentially provides a spatially averaged estimate of standard error [14]. The RtGCV was 2.7% for 1951–1980 and 3.3% for 1981–2010 as a percentage of the surface mean (Table 2).

### 3.5 Limitations

Most stations were missing observations for at least some portion of the period considered for this study. With this data report, we published the number of years of data upon which the calculations are based to allow users to make decisions about the use of this dataset. Future work will consider the use of fully in-filled time series for a thirty-year period using estimates for missing HDD values. Canadian in situ stations were concentrated in southern latitudes. Notably much of northern Canada is monitored through a relatively sparse network. To address this feature of the datasets, ANUSPLIN predictions were evaluated for a set of 60 stations selected to better reflect the range in latitude, longitude, and elevation across the country [12].

## Ethics Statement

This work did not involve human subjects or experiments using animals.

## CRediT Author Statement

**Heather MacDonald:** Conceptualization, Methodology, Formal Analysis, Validation, Writing -Original draft preparation, Writing - review & editing.  **John Pedlar:** Conceptualization, Methodology, Visualization, Formal Analysis, Validation, Writing –Original draft preparation, Writing - review & editing. **Daniel McKenney:** Conceptualization, Methodology, Writing –Original draft preparation, Writing - review & editing. **Kevin Lawrence:** Data curation, Investigation, Validation. **Kaitlin de Boer:** Data curation, Investigation, Visualization, Investigation, Writing - review & editing. **Michael Hutchinson:** Software, Methodology, results validation and review, manuscript editing.

## Declaration of Competing Interest

The authors declare that they have no known competing financial interests or personal relationships that could have appeared to influence the work reported in this paper.

## Data Availability

Heating Degree Days Canada 1951-1980, 1981-2010 (Original data) (Open Science Framework).
